# Spermatozoa Proteins Involved in ROS Generation and Antioxidant Defense Are Differentially Acetylated in Idiopathic Infertility

**DOI:** 10.3390/antiox14121410

**Published:** 2025-11-26

**Authors:** Lisa Goutami, Anwesha Pradhan, Ajaya Kumar Moharana, Soumya Ranjan Jena, Manesh Kumar Panner Selvam, Suresh C. Sikka, Luna Samanta

**Affiliations:** 1 Redox Biology & Proteomics Laboratory, Department of Zoology and Centre of Excellence for Environment and Public Health, Ravenshaw University, Cuttack 753003, India; goutamilisa.95@gmail.com (L.G.); anweshapradhan5098@gmail.com (A.P.); srjenaa@gmail.com (S.R.J.); 2 Department of Urology, Tulane University School of Medicine, New Orleans, LA 70112, USA; mpannerselvam@tulane.edu

**Keywords:** idiopathic male infertility (IMI), lysine acetylation, acetyl-proteomics, oxidative stress, SOD1, PARK7, PRKACA

## Abstract

Idiopathic male infertility (IMI), which accounts for nearly 50% of male infertility cases, remains a major clinical challenge due to the inability of standard semen analysis to reveal underlying molecular defects. Post-translational modifications such as lysine acetylation are increasingly recognized as key regulators of sperm function, affecting antioxidant defense, energy metabolism, and spermatogenesis. In this study, global acetyl-proteomic profiling of spermatozoa from idiopathic infertile patients (IIP) and fertile donors (FD) was performed using immunoprecipitation-based enrichment followed by high-resolution LC–MS/MS. Bioinformatics analyses, including STRING, Cytoscape, and Ingenuity Pathway Analysis (IPA), identified 718 differentially acetylated proteins (DAPs), with significant enrichment in pathways related to redox homeostasis, molecular transport, glycolysis, and mitochondrial metabolism. Hub proteins such as SOD1, PARK7, and PRKACA emerged as key regulators linking oxidative stress with defective motility and impaired sperm physiology. Western blot validation confirmed the downregulation of these hub proteins in IIP, supporting their role in redox imbalance and mitochondrial dysfunction. Our findings highlight dysregulated lysine acetylation as a defining molecular feature of IMI and suggest that targeting acetylation-associated pathways may provide novel diagnostic biomarkers and therapeutic strategies to improve sperm function and male reproductive outcomes.

## 1. Introduction

Spermatozoa are highly specialized cells that lack transcriptional and translational machinery, rendering them uniquely dependent on post-translational mechanisms for functional regulation. This dependence makes them particularly vulnerable to alterations in protein integrity, which can compromise motility, viability, and fertilizing ability. Molecular chaperones, especially heat shock proteins, play key roles in maintaining proteostasis and redox balance under such constraints [1].

Oxidative stress is a major determinant of male reproductive dysfunction, arising from an imbalance between reactive oxygen species (ROS) production and antioxidant defenses [2,3,4]. While physiological ROS levels are essential for sperm maturation and capacitation, excessive ROS induce oxidative damage and apoptosis [5,6]. Superoxide anions, hydrogen peroxide, and hydroxyl radicals can modify sperm lipids, proteins, and DNA, leading to impaired motility and fertilization potential [5]. Elevated ROS levels are particularly implicated in idiopathic asthenozoospermia [6].

Given their minimal cytoplasmic volume, mammalian spermatozoa exhibit limited antioxidant defenses, increasing susceptibility to ROS-induced injury [7]. Enzymatic antioxidants such as superoxide dismutase (SOD), catalase (CAT), and glutathione peroxidase (GPx) act sequentially to neutralize ROS, with non-enzymatic antioxidants including vitamins C and E providing additional protection [4,8,9]. The maintenance of redox equilibrium is therefore critical for sperm function and fertility [4].

Among post-translational regulatory systems, lysine acetylation has emerged as an important modulator of protein stability, activity, and interaction networks. Governed by lysine acetyltransferases (KATs) and deacetylases (KDACs), this reversible modification regulates molecular chaperones and antioxidant enzymes. In spermatozoa, acetylation modulates the activity of heat shock protein A2 (HSPA2), a central regulator of redox homeostasis [1]. Aberrant acetylation profiles have been reported in sperm from infertile men, with HSPA2 identified as a potential biomarker of idiopathic infertility [1]. Dysregulated acetylation may impair antioxidant defense, enhance ROS generation, and exacerbate oxidative stress, thereby contributing to the pathogenesis of idiopathic male infertility [5,10,11].

Oxidative stress further disrupts spermatogenesis through mitochondrial dysfunction and activation of apoptotic cascades, including Trx–ASK1–Bax signaling and cytochrome c release, culminating in reduced sperm concentration and quality [5]. ROS-mediated signaling via calcium and MAPK pathways also regulates antioxidant enzyme expression and redox homeostasis [4,5,12].

Despite growing evidence of oxidative stress and acetylation-mediated regulation in sperm function, the mechanistic link between lysine acetylation and redox imbalance in idiopathic male infertility (IMI) remains poorly understood. This study aims to identify acetylation-dependent molecular alterations that impair redox homeostasis and sperm function in IMI through global acetylproteomic profiling. The findings are expected to provide mechanistic insights into the acetylation–redox interface in sperm biology and identify novel molecular targets for diagnostic and therapeutic intervention in male infertility.

## 2. Materials and Methods

### 2.1. Ethics Statement and Patient Selection

Following approval from the Institutional Ethics Committee, patients attending the infertility clinic and verified control (fertile donors) at Kar Clinic and Hospital Pvt. Ltd., Bhubaneswar, Odisha, India, were recruited for the study. Informed written consent was obtained from all individuals prior to inclusion in the study. Exclusion criteria included leukocytospermia (Endtz positive), azoospermia, systemic illnesses, reproductive tract inflammations (orchitis, epididymitis, urethritis, testicular atrophy), diabetes, sexually transmitted infections, varicocele, and ongoing medication. The patient cohort comprised individuals presenting with at least one abnormal semen parameter according to WHO 2021 guidelines: sperm concentration (<16 × 10^6^/mL), motility (<42%), vitality (<54%), or normal morphology (<4%). Only men under 40 years of age, non-smokers, non-alcoholic, and with a normal body mass index (BMI) were considered. The control group consisted of healthy individuals with no known medical conditions and of proven fertility within the past year, without any history of pregnancy loss. Sperm proteome profiling was conducted in accordance with the Minimum Information about a Proteomics Experiment (MIAPE) recommendation of the Human Proteome Organization–Proteomics Standards Initiative (HUPO-PSI). To account for biological variation, two individual samples and one pooled sample (prepared from ten randomly chosen participants) were analyzed. Each experiment was performed in triplicate to minimize technical variability. The adequacy of the sample size was assessed following the statistical framework proposed by Clough et al. [13] for high-throughput label-free LC–MS/MS studies and supported by comparable research designs. Pooled samples were generated by equalizing both protein concentration and sperm count from each donor to ensure consistent representation [13].

### 2.2. Sample Preparation

Semen samples were collected from all participants in both groups (idiopathic infertile patients, *n* = 57; fertile donors, *n* = 43) following 3–5 days of sexual abstinence via masturbation. Each sample was incubated at 37 °C for 20–30 min to ensure complete liquefaction before assessment, which was carried out in accordance with the 2010 & 2021 World Health Organization (WHO) guidelines [14]. The basic semen analysis included evaluation of macroscopic parameters (volume, pH, color, viscosity, and liquefaction time) and microscopic parameters (sperm concentration, motility, and morphology). In addition, the peroxidase (Endtz) test was performed to screen for leukocytospermia. Samples showing >1.0 × 10^6^/mL round cells with a positive peroxidase test were excluded. Following liquefaction and initial semen analysis, samples were centrifuged at 400× *g* for 20 min at 37 °C to separate spermatozoa from seminal plasma.

### 2.3. Sperm Protein Extraction and Tryptic Digestion

After separation of the sperm pellet from seminal plasma, the pellet was washed three times with phosphate-buffered saline (PBS) and centrifuged at 1000× *g* for 10 min at 4 °C. The resulting pellet was resuspended in radio-immunoprecipitation assay (RIPA) buffer supplemented with a protease inhibitor cocktail (Complete ULTRA Tablets, Roche India, Mumbai) and incubated overnight at 4 °C to ensure complete lysis. The lysate was then centrifuged at 14,000× *g* for 30 min at 4 °C, and the supernatant containing soluble sperm proteins was collected. Protein concentration was quantified using the bicinchoninic acid (BCA) assay. The homogenates were diluted in 50 mM ammonium bicarbonate (NH_4_HCO_3_), reduced with 1 mM 1,4-dithiothreitol (DTT) for 30 min, and alkylated in the dark with 5 mM iodoacetamide for 30 min. Proteins were digested overnight at room temperature with trypsin (Promega Corporation, Maddison, WI, USA) at a 1:50 enzyme-to-substrate ratio, maintained at pH 7.8. Following digestion, peptides were acidified using 1% formic acid and 0.1% trifluoroacetic acid (TFA), desalted and purified on Sep-Pak C18 cartridges (Waters), and finally eluted with 50% acetonitrile.

### 2.4. Immunoprecipitation of Lysine-Acetylated Peptides of Spermatozoa

The lysine-acetylated peptides were affinity purified in triplicates. The tryptic digested samples were treated in duplicate with a pre-washed antibody-bead slurry [Acetylated Lysine Monoclonal Antibody (1C6) conjugated agarose beads, abcam] at 4 °C overnight with mild agitation in order to obtain lysine-acetylated peptides. The beads were washed four times with Wash buffer [10 mM Tris (pH 7.4), 1 mM EGTA (pH 8.0), 1 mM EDTA, 1% Triton X-100, 150 mM NaCl, 0.2 mM Na3VO4 and Protease inhibitor cocktail]. The non-denaturing 0.1–0.2 M glycine buffer at pH 2.6 was used to elute the bound proteins from the beads. The elution process was performed twice. Upon pooling the eluates, an equivalent volume of Tris pH 8.0 was added to neutralize the pH. The resulting acetylated lysine-enriched peptides were reconstituted in buffer containing 2% acetonitrile and 0.1% formic acid after being purified further using reversed-phase Sep-Pak C18 columns (Waters).

### 2.5. Proteome Profiling of Immunoprecipitated Lysine Acetylated Peptides

LC-MS/MS was used to perform proteomic analysis on immunoprecipitated acetylated peptides. A self-packed 75 μm × 25 cm Picofrit emitter (New Objective Inc., Woburn, MA, USA) was loaded with the acetylated peptides from each sample (*n* = 3 per group, consisting of one pooled sample and two individual samples), and the eluted peptides were collected using a liquid chromatography system. The gradient consisted of a linear ramp from 3 to 42.5% buffer B (buffer A: 0.1% formic acid in water; buffer B: 0.1% formic acid in 80% acetonitrile) for 105 min, followed by ramps of 20 min to 60% buffer B, 5 min to 99% buffer B, and 10 min to flush at 99% buffer. All flowrates were kept constant at 300 nL/min. The total lysate peptides were eluted by escalating from 3 to 50% buffer B during a 105 min gradient, then ramping to 80% buffer B for 20 min, 99% buffer B for 5 min, and flushing at 99% buffer B for 10 min. All of the peptide ions were captured using a Fusion Orbitrap mass spectrometer in high-speed mode with a 5 s cycle time. Full scans (scan range: 400 to 1600 *m*/*z*) were collected at 120,000 resolutions with an automated gain control setting of 200,000 and a maximum injection time of 50 ms. Rapid scan speed and maximum injection period of 35 ms were used to acquire higher energy collision dissociation (HCD) tandem mass spectra in the ion trap. Dynamic exclusion was set to 20 s, the collision energy was set to 30%, and only ions with charge states between 2 and 7 were collected.

### 2.6. Label-Free Quantification (LFQ) of Acetylation Sites

The raw data for the whole lysine acetylated proteome were analyzed using MaxQuant’s integrated Andromeda search engine (version 1.5.2.8) and compared to the UniProt HUMAN reference proteome (HUPO) database. The protease, trypsin/P, was recruited as having selectivity for cleaving “K/R” at its C-terminus, unless “P” comes before it, and allowing up to two miscleavages. A fixed modification of cysteine carbamidomethylation (+57.0215 Da) and variable modifications of methionine oxidation (+15.9949 Da) and N-terminal acetylation (+42.0106 Da) were assigned. The lysine-specific acetylation (+42.0106 Da) was identified as a variable modification for the acetylated lysine-enriched peptide screening. The acetylome database search allowed global proteins to have a default of two miscleavages, while tryptic peptides with up to five miscleavages were included. To ensure a high degree of confidence in protein identification, the false discovery rate (FDR) for peptides and spectral matches was set at 0.01. The false discovery rates (FDRs) for proteins, site decoy fraction, and peptide spectral matches were all 1%. The MaxQuant algorithms computed summary statistics by recognizing and scoring peptide peaks, performing mass calibration, and measuring proteins. The protein abundances were log-transformed (Log2) for additional analysis after being adjusted using the LFQ method. Peptide ratios were measured using the label-free method by evaluating the intensities of detected peptides and estimating the abundance of each protein by calculating the median ratio values.

### 2.7. Bioinformatics Analysis

Differentially expressed proteins (DEPs) containing acetyl-lysine sites were identified based on a log_2_ fold change ≥|1.0|≤ and Welch’s *t*-test *p*-value < 0.05. The DEPs were subjected to functional annotation and enrichment analyses using publicly available bioinformatics resources, including STRING, UniProt, and Cytoscape. Enrichment terms were prioritized according to *p*-values (hypergeometric test) via the ClueGO plugin in Cytoscape. Protein distribution was visualized using Venn diagrams generated with Venny 2.1. Hierarchical clustering of DEPs between fertile donors and idiopathic infertile patients was performed using the R package (version 3.4.4, ComplexHeatmap library). Clustering was based on a Euclidean distance correlation matrix, and the resulting dendrograms highlighted patterns of protein expression and interaction. Overrepresented Gene Ontology (GO) categories were identified with the BiNGO plugin in Cytoscape, providing insight into the functional significance of the altered proteins. To explore canonical pathways, significantly acetylated proteins were analyzed using Ingenuity Pathway Analysis (IPA; QIAGEN, Redwood City, CA, USA). Proteins were uploaded into IPA, and the most significantly enriched canonical pathways were reported along with *p*-values calculated using a right-tailed Fisher’s exact test. Across all analyses, a *p*-value < 0.05 was considered statistically significant.

### 2.8. Protein–Protein Interaction (PPI) Network Construction and Hub Lysine Acetylated Protein Identification

The STRING database (version 12.0) (http://string-db.org/) was used to analyze the network of protein–protein interactions (PPIs). Cytoscape software (version 3.9.1) was used to visualize the resulting network. The “Analyze Network” tool (version 3.9.1) was used to generate node degree values, highlighting nodes with a degree of ≥5. The CytoHubba plug-in was employed to identify the top 10 hub genes for each of the four algorithms: Degree, DMNC (Density of Maximum Neighborhood Component), MCC (Maximal Clique Centrality) and MNC (Maximum Neighborhood Component).

### 2.9. Western Blotting

The expression of the key hub proteins was validated by Western blot. The following primary antibodies were used for immunodetection, viz, anti-human Superoxide Dismutase 1/SOD1 Antibody (sc-17767, mouse, Santa Cruz, Dollas, TX, USA), PARK7 Mouse Monoclonal Antibody (BF9398, Affinity Biosciences, Cincinnati, OH, USA), PKA alpha/beta/gamma CAT Antibody (AF7746, Rabbit, Affinity Biosciences, Cincinnati, OH, USA) and AKAP4(DF10048, Affinity Biosciences, Cincinnati, OH, USA). Triplicate tests were performed on two individual and one pooled sample from each group to maintain biological and technical variability. The protein concentration of each sample was normalized in each group. Spermatozoa were lysed in RIPA lysis solution (Sigma-Aldrich, St. Louis, MO, USA) supplemented with a protease inhibitor cocktail (Roche, Indianapolis, IN, USA) and incubated overnight at 4 °C. Protein samples (20–30 μg per well) were separated using 4–20% SDS-PAGE gels and subsequently transferred to polyvinylidene difluoride (PVDF) membranes. The membranes were incubated for 2 h with 5% non-fat dry milk in Tris-buffered saline with Tween 20 (TBST). The membranes were then incubated overnight at 4 °C with respective primary antibodies (anti-human SOD1, PARK 7, PRKACA and AKAP4 antibody), followed by 3 h incubation at room temperature with the corresponding secondary antibodies Peroxidase conjugated Goat anti-Mouse IgG (H + L) (11-301) and Peroxidase conjugated Goat anti-Rabbit IgG (H + L) (11-315). The membranes were rinsed with TBST, and protein bands were detected utilizing the Pierce™ ECL Western Blotting Substrate (Thermo Scientific, Rockford, IL, USA) on a ChemiDoc™ MP Imaging System (BioRad, Hercules, CA, USA). Densitometric analysis of the Western blot images was conducted utilizing Image Lab 6.0.1 (BioRad Laboratories, Gurugram, India), with results normalized via the total intensity method. Data were presented as fold changes in relation to the control (fertile donor) group.

### 2.10. Statistical Analysis

Statistical analysis was conducted using MedCalc Statistical Software, version 17.4 (MedCalc Software, Ostend, Belgium). Data are presented as mean ± SD. Levene’s test was used to determine the homogeneity of variance, and the Shapiro–Wilk test was used to determine whether the data distribution was normal. Sperm parameters were analyzed using the Mann–Whitney U-test. The LC-MS/MS proteomics and Western blotting findings were subjected to the Welch’s *t*-test (*t*-test for unequal variances). Statistically significant differences were identified using a *p*-value of less than 0.05. All statistical bioinformatics analyses were carried out using R (https://www.r-project.org/, 4.2.1 version).

## 3. Results

All idiopathic infertile patients (IIP) participating in this study exhibit at least one semen parameter in semen analysis that falls below the criteria established by WHO (WHO 2010). Despite the average values falling within the normal range, sperm concentration, motility, morphology, and vitality exhibited significant differences compared to the control (fertile donors) (Supplementary Table S1).

### 3.1. Proteomic Analysis of Lysine Acetylated Spermatozoa Proteins

**Figure 1:**
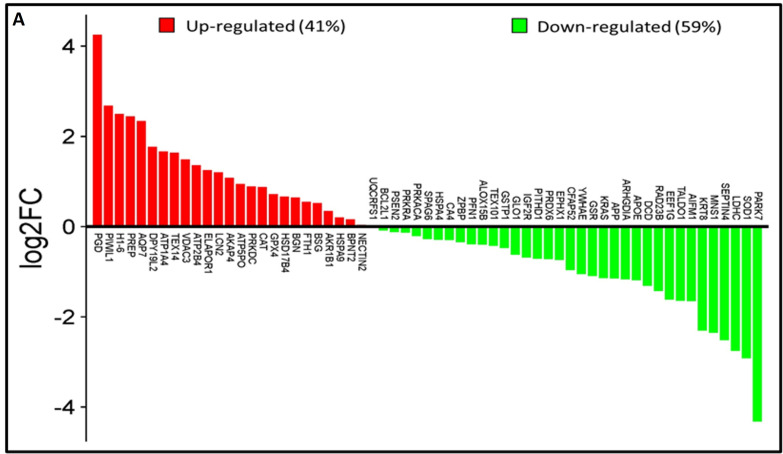

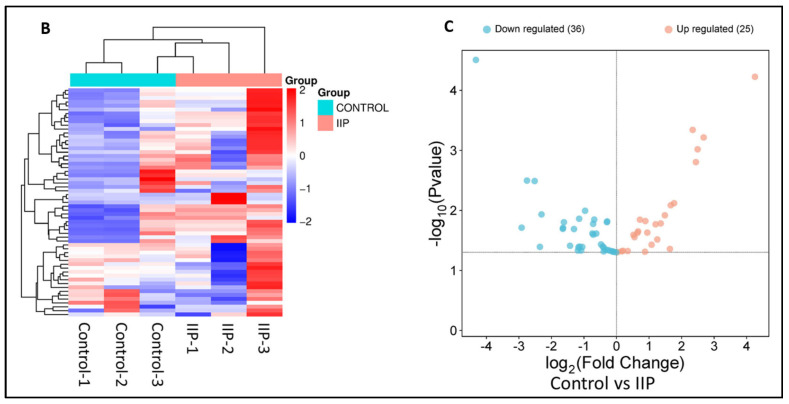
Comparative global lysine acetyl-proteomic profiling of spermatozoa proteins between Control (fertile donors) and idiopathic infertile patients (IIP). (**A**) Bar graph showing thelog_2_ fold change of lysine-acetylated differentially expressed proteins (DEPs) in idiopathic infertile patients with respect to control (FD) (Red bars: Up Regulated proteins; Green Bars: Down Regulated proteins). (**B**) Heat map with hierarchical clustering of lysine-acetylated proteins. Column clustering segregated control and IIP groups, while row clustering separated overexpressed DEPs in IIP from underexpressed DEPs. Blue and red indicate low and high expression levels, respectively (see scale bar). (**C**) Volcano plot of lysine-acetylated stress proteins in control versus IIP spermatozoa. Red and blue dots indicate significantly altered proteins with both large fold changes (*x*-axis) and high statistical significance (−log10 *p* value, *y*-axis). The horizontal dashed line marks the significance threshold (*p* ≤ 0.05), while vertical dashed lines indicate fold-change cutoffs (−1.0 ≤ FC ≥ 1.0), identifying over-expressed (right) and under-expressed (left) proteins.

Analysis of the lysine acetyl-proteome of spermatozoa from idiopathic infertile patients (IIP) and control (fertile donors) identified 2988 proteins with potential lysine acetylation sites, showing significant differential expression. Among these, 718 DEPs (log_2_ fold change ≥|1.0|≤ and Welch’s *t*-test *p*-value < 0.05) were common to both groups. Notably, 61 lysine acetylated DEPs (25 upregulated and 36 downregulated) were associated with ROS metabolism and antioxidant defense (Figure 1A, Supplementary Table S2). Hierarchical clustering by heatmap clearly distinguishes over- and under-expressed lysine acetylated proteins between the two groups (Figure 1B). Furthermore, the volcano plot (Figure 1C) highlighted 25 upregulated and 36 downregulated ROS and antioxidant-related lysine acetylated proteins in IIP compared to FD (*t*-test, *p* ≤ 0.05, fold change ≥|1.0|≤).

### 3.2. Distribution Pattern and Functional Annotation of Differentially Expressed Lysine-Acetylated Proteins of Spermatozoa

**Figure 2:**
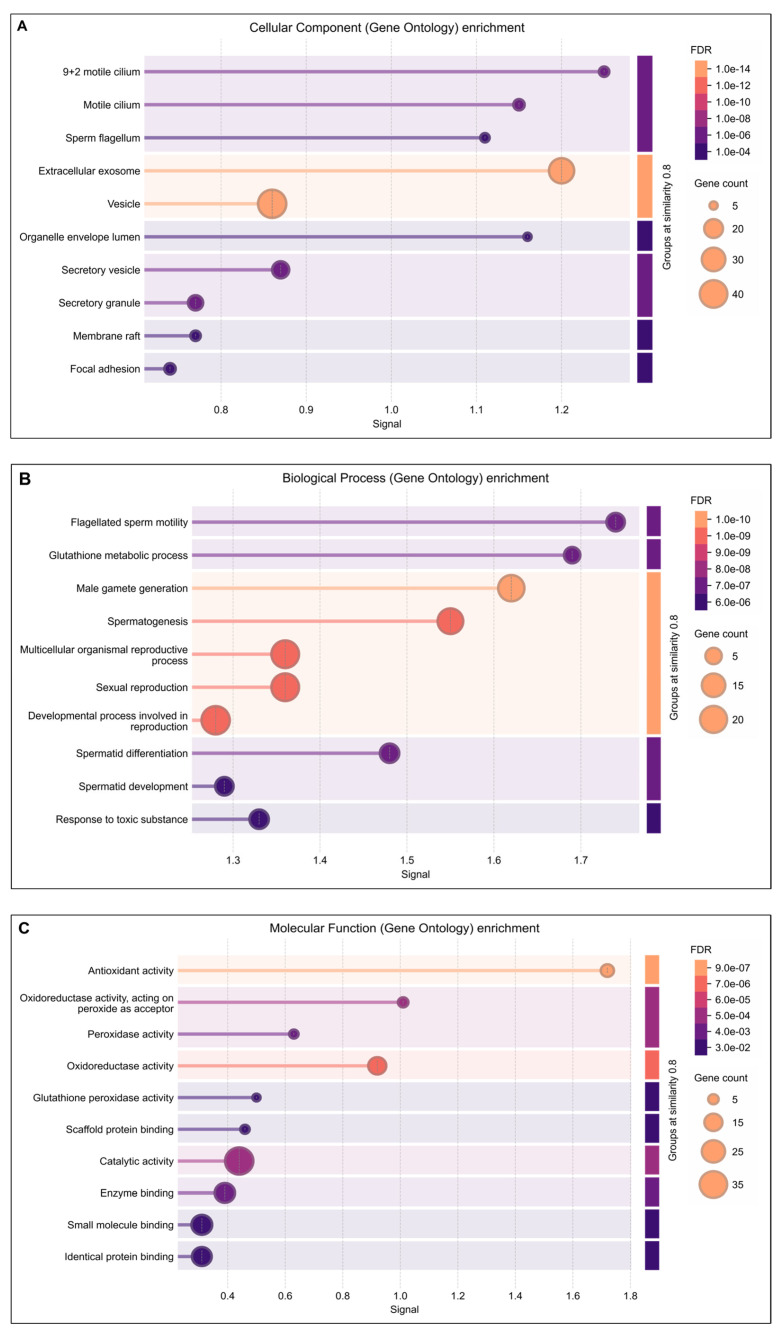
DAVIDGene Ontology (GO) enrichment analysis result of differentially expressed lysine acetylated proteins in IIP: Idiopathic infertile patients compared to control: fertile donor. The size of dots showing gene counts, length of line represents signal and color of line graph showing the false discovery rate of the top GO terms for (**A**) cellular component, (**B**) biological process, and (**C**) molecular function and *p* < 0.05.

Functional annotation using advanced bioinformatics resources, including GO Term Finder, UniProt, and DAVID, demonstrated that several lysine-acetylated proteins are implicated in ROS-mediated oxidative stress regulation and antioxidant-related multifunctional pathways. In this study, GO cellular component analysis indicated enrichment of acetylated proteins in the motile cilium, sperm flagellum, extracellular exosomes, and secretary vesicles (Figure 2A). The biological process analysis highlighted significant involvement of these proteins in flagellated sperm motility, glutathione metabolism, spermatogenesis, spermatid differentiation and development, and response to toxic substances (Figure 2B). Furthermore, the molecular function analysis revealed strong associations with antioxidant activity, oxidoreductase activity, peroxidase activity, and glutathione peroxidase activity (Figure 2C).

### 3.3. Pathway Analysis of Lysine Acetylated Spermatozoa Differential Expressed Proteins

**Figure 3:**
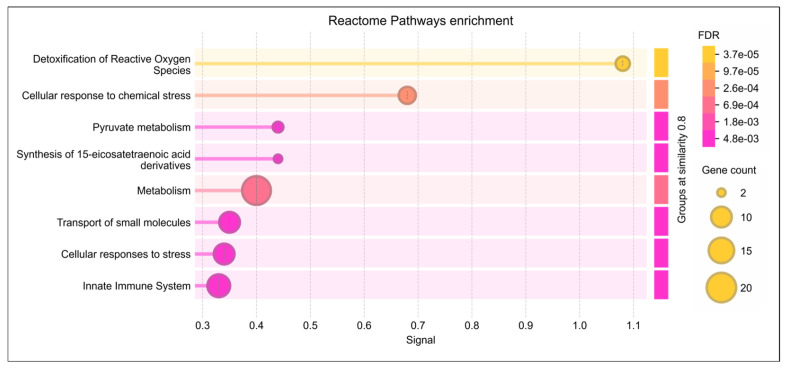
Reactome pathway enrichment analysis of lysine-acetylated differentially expressed proteins (DEPs) in idiopathic infertile patients (IIP) compared to fertile donors (FD). The dot plot shows the top significantly enriched Reactome pathways associated with the acetylated DEPs. The *x*-axis represents the gene ratio (proportion of DEPs involved in each pathway), while the *y*-axis lists the enriched pathways. The size of each dot indicates the number of DEPs mapped to the pathway (gene count), and the color gradient represents the statistical significance based on the False Discovery Rate (FDR), with darker shades indicating higher significance. Key enriched pathways include detoxification of reactive oxygen species, cellular response to stress, and pyruvate metabolism, highlighting the role of dysregulated protein acetylation in redox homeostasis and energy metabolism in idiopathic male infertility.

Reactome pathway analysis of the lysine-acetylated DEPs revealed significant dysregulation of pathways related to detoxification of ROS, cellular response to stress, and pyruvate metabolism (*p* ≤ 0.05). These DEPs were also strongly associated with oxidative stress regulation and antioxidant defense mechanisms (Figure 3). Further analysis using IPA identified their involvement in free radical scavenging and molecular transport (Table 1). Within the reproductive system disease pathway, IPA highlighted a strong link between these DEPs in infertility as well as asthenozoospermia (*p* < 0.05) (Table 1). Moreover, under the diseases and disorders category, enriched functions were predominantly associated with reproductive diseases and infertility.

### 3.4. Protein–Protein Interaction Network for Identification of Lysine-Acetylated Hub Proteins

**Figure 4:**
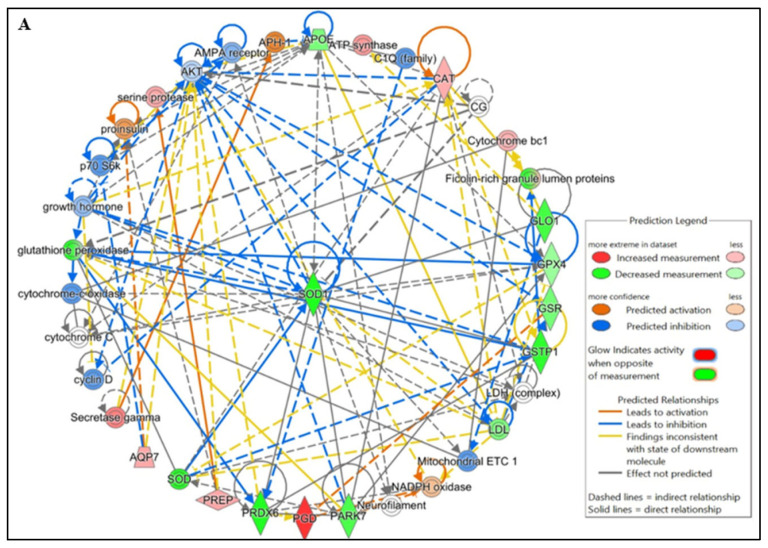

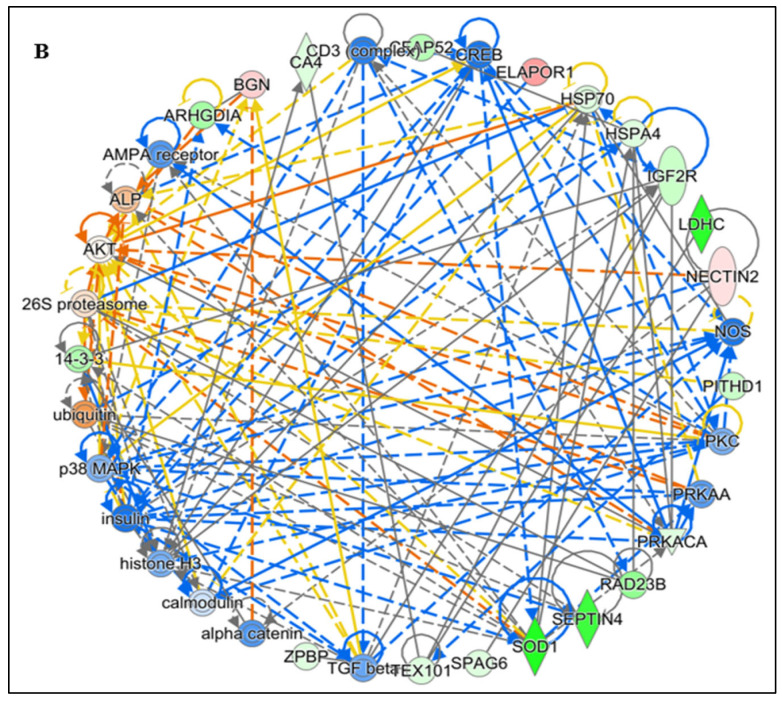
Functional Analysis of the top (**A**) Free Radical Scavenging and Molecular Transport and (**B**) Reproductive system diseases Network by Ingenuity Pathway Analysis (IPA) software (https://digitalinsights.qiagen.com/IPA, accessed on 14 October 2025) of the differentially expressed lysine acetylated proteins (DEPs) from IIP group with respect to control group.

IPA pathway analysis revealed that protein–protein interactions (PPIs) among lysine-acetylated DEPs involved in free radical scavenging and molecular transport, which were further associated with infertility and asthenozoospermia in idiopathic infertile conditions. Notably, many of these acetylated proteins were directly related to sperm function and acetylation-mediated regulation. To strengthen these findings, IPA-based PPI network analysis was conducted using DEPs from IIP and control spermatozoa (Figure 4A,B).

**Figure 5:**
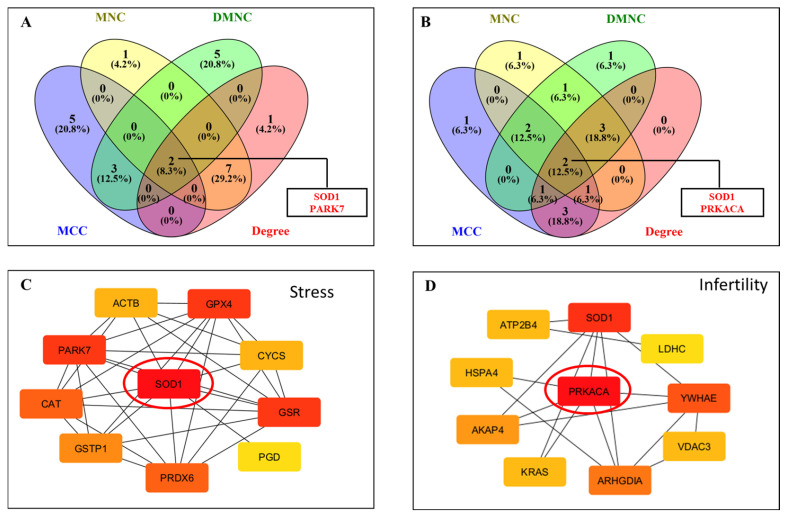
Construction of protein–protein interaction (PPI) networks of lysine-acetylated proteins in idiopathic infertile patients compared with controls. (**A**) Venn diagram analysis showing overlapping differentially expressed proteins (DEPs) identified by four CytoHubba algorithms (MNC, MCC, DMNC, and Degree) in network 1. Two hub proteins, SOD1 and PARK7, were identified. (**B**) Venn diagram analysis showing overlapping DEPs identified by the same four algorithms in network 2. Two hub proteins, PRKACA and SOD1, were identified. (**C**) PPI network highlighting SOD1 as a central hub protein interacting with key antioxidant proteins. (**D**) PPI network highlighting PRKACA as a central hub protein interacting with proteins involved in energy metabolism and signaling.

Hub gene identification through CytoHubba was performed with four topological algorithms—Degree, MNC (Maximum Neighborhood Component), MCC (Maximal Clique Centrality), and DMNC (Density of Maximum Neighborhood Component). The top 10 hub genes from each method were compared, and intersecting results identified SOD1 and PARK7 as common hubs in the free radical scavenging and molecular transport network (Figure 5A,C). Likewise, in the infertility and asthenozoospermia disease network, PRKACA and SOD1 emerged as shared hub proteins (Figure 5B,D). The IPA network showed the involvement of these differentially acetylated hub proteins to have their roles in antioxidant defense, sperm maturation, motility, and survival, underscoring the pivotal biological impact of lysine acetylation in regulating male fertility.

### 3.5. Expression Profile of Key Hub Proteins

**Figure 6:**
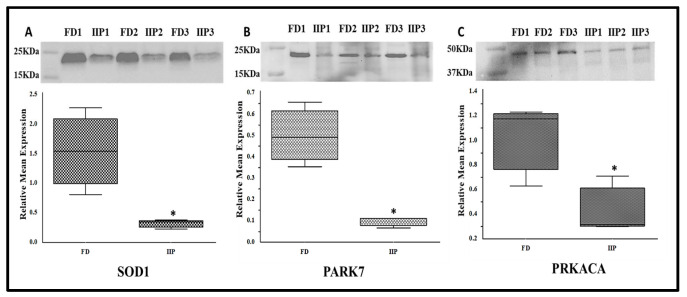
Representative Western blot of hub proteins and corresponding densitometry analysis in spermatozoa of FD: fertile donor (*n* = 3) and IIP: idiopathic infertile patient (*n* = 3) with total protein normalization (in arbitrary unit). * *p* < 0.05 with respect to the control patients. (**A**) SOD1 (**B**) PARK 7 (**C**) PRKACA.

The key hub proteins predicted in the top hub pathway were SOD1, PARK 7 and PRKACA. The immune-precipitated hub proteins depicting the expression of lysin acetylation were validated by Western blot which corroborated the LC-MS/MS data. These proteins were found to be under expressed in the IIP group compared to the FD group (Figure 6). Since the molecular weight of these proteins was similar on Western blot, the down-regulation may be due to overall protein expression than differential-lysine acetylation.

**Figure 7:**
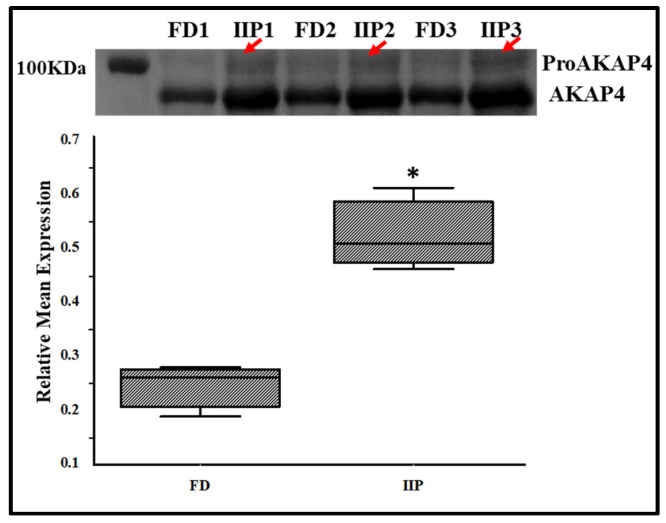
Representative Western Blot of AKAP4 and corresponding densitometry analysis in spermatozoa of control (C): fertile donor (FD) (*n* = 3) and IIP: idiopathic infertile patient (*n* = 3) with total protein normalization (in arbitrary unit). * *p* < 0.05 with respect to the control patients.

To further validate the LC-MSMS data, the expression profile of the immunoprecipitated up-regulated protein AKAP4, a scaffold protein that is crucial for sperm motility by anchoring enzymes to the sperm’s fibrous sheath was studied by Western blot analysis, which corroborated the LC–MS/MS findings (Figure 7). In IIP group retention of a higher molecular weight band (100 KDa) corresponding to proAKAP4 was visualized which was not recognizable in the donor group (Figure 7, red arrows).

## 4. Discussion

Idiopathic male infertility (IMI) accounts for nearly half of all male infertility cases and remains a major clinical challenge, as standard semen analysis often fails to detect the underlying molecular defects [15,16]. Because mature spermatozoa are transcriptionally and translationally inactive, their functional competence depends largely on post-translational modifications (PTMs) such as phosphorylation, ubiquitination, and acetylation [16,17]. Lysine acetylation, in particular, plays a critical role in regulating mitochondrial metabolism, chromatin remodeling, and antioxidant defense. The present global acetyl-proteomic analysis of spermatozoa from idiopathic infertile patients (IIP) reveals extensive acetylation changes associated with oxidative stress, energy metabolism, and structural maturation, supporting a central role of acetylation-dependent protein regulation in maintaining sperm function.

### 4.1. Redox Dysregulation, Antioxidant Defense, and Proteostasis

Ingenuity Pathway Analysis (IPA) indicated that differentially acetylated proteins (DAPs) were highly enriched in free radical scavenging pathways, molecular transport, and reproductive system disorders (*p* < 0.05). The principal protein network included several antioxidant enzymes—SOD1, PRDX6, CAT, GPX4, and PARK7 (DJ-1)—along with glutathione pathway members GSTP1, GSS, and GSTM1, all of which are essential for neutralizing ROS. Dysregulated acetylation of these enzymes may compromise their catalytic efficiency, heightening susceptibility to oxidative stress-induced lipid peroxidation and DNA damage. Prior studies demonstrate that enzymes such as SODs, PRDXs, CAT, and GPX4 contain acetylation sites that influence their activity and redox regulatory functions [18,19], consistent with the reduced acetylation observed in our dataset.

Proteostasis is also impacted under oxidative conditions, where the ubiquitin–proteasome pathway (UPP) and molecular chaperones coordinate protein folding and degradation [20]. Our earlier work demonstrated disrupted acetylation of 26 chaperone proteins concomitant with elevated 4-hydroxynonenal (4-HNE) levels, strongly suggesting that oxidative stress interacts with acetylation-dependent chaperone regulation to impair protein quality control [1]. Such disturbances may contribute to sperm deformity, impaired motility, and functional decline in IMI.

### 4.2. Energy Metabolism, Motility Regulation, and Structural Integrity

A second major acetylation network involved proteins regulating energy production, signal transduction, and motility, including PRKACA, PRKAA, AKAP4, AKT, MAPK, LDHC, calmodulin, and HSP70 family members. These molecules orchestrate glycolysis, oxidative phosphorylation, capacitation, and flagellar dynamics. Site-specific lysine acetylation enhances activity of several metabolic kinases—for example, K62 acetylation increases PKA activity [21]—and proteins such as LDHC and PRKACA become acetylated during capacitation but not in non-capacitated sperm [19]. Altered acetylation in IIP therefore likely disturbs ATP generation and associated phosphorylation cascades, contributing to reduced motility.

AKAP4, a principal architectural element of the sperm fibrous sheath, plays a key role in cAMP/PKA signaling required for motility. Overexpression and hyperacetylation of AKAP4 and its precursor proAKAP4 in IIP suggest altered structural or regulatory interactions. Previous evidence shows that both AKAP4 forms are targets of 4-HNE adduction in spermatids and mature sperm [22], and 4-HNE exposure leads to AKAP4 degradation and aggregation, reducing capacitation-related phosphorylation [22]. Because 4-HNE adducts form at cyc, his, and lys residues [23], oxidative stress may facilitate aberrant hyperacetylation by inhibiting SIRT3/SIRT1 deacetylases. Given proAKAP4’s role as a reservoir maintaining motility and capacitation [24], redox-driven acetylation changes in AKAP4 could compromise axonemal stability and ATP utilization, linking oxidative imbalance to idiopathic infertility.

DAPs were also enriched in pathways associated with glycolysis, pyruvate metabolism, and mitochondrial oxidative phosphorylation, all fundamental to sperm motility and fertilization success [25]. Moreover, acetylation changes in proteins involved in spermatid differentiation, flagellar assembly, and axonemal organization suggest impaired structural maturation as an additional mechanism contributing to IMI.

### 4.3. Key Regulatory Proteins in Redox and Metabolic Homeostasis

SOD1: A primary antioxidant enzyme that catalyzes the dismutation of superoxide radicals and plays an essential role in maintaining sperm motility and fertilization capacity [16,26,27]. Reduced SOD1 activity correlates with poor reproductive outcomes [28,29]. Aberrant acetylation identified here may diminish catalytic efficiency, exacerbating redox imbalance in spermatozoa.

PRKACA: This catalytic subunit of PKA governs motility and capacitation via the phosphorylation of motility-related proteins [30]. Altered acetylation could impair its enzymatic activity or disrupt its association with regulatory subunits, contributing to asthenozoospermia and defective capacitation [31].

PARK7 (DJ-1): PARK7 is a multifunctional redox-sensitive chaperone that stabilizes SOD1 and PRDXs and maintains mitochondrial homeostasis [17,32]. Acetylation changes in PARK7 may weaken its antioxidant and cytoprotective roles, increasing sperm vulnerability to oxidative injury and linking infertility with other redox-associated diseases [32].

### 4.4. Epigenetic, Clinical, and Therapeutic Implications

Several DAPs overlapped with pathways implicated in reproductive disorders and cancer, highlighting the broad biological significance of lysine acetylation in cell fate regulation. Aberrant acetylation during spermiogenesis may interfere with chromatin compaction and protamine–histone exchange, compromising genome integrity [33]. These findings reveal an epigenetic component of IMI that extends beyond conventional semen parameters.

Therapeutic strategies targeting oxidative and acetylation imbalance hold promise. Various antioxidants such as resveratrol, anthocyanins, α-tocopherol, zinc, D-aspartate, and coenzyme Q10 have been shown to reduce ROS-mediated damage and improve semen quality [34,35,36,37,38]. Complementary proteomic analyses from our group demonstrated that antioxidants modulate redox defense pathways and key sperm proteins such as NDUFS1, CCT3, PRKAR1A, and SPA17, supporting their potential application as biomarkers for treatment monitoring [39].

In summary, dysregulated protein acetylation emerges as a defining molecular feature of idiopathic male infertility, disrupting antioxidant defense, metabolic efficiency, and sperm structural maturation. While informative, the lack of pregnancy outcome data and limited sample size constrain generalizability. Future longitudinal studies integrating proteomics, sperm function tests, and reproductive outcomes including pregnancy and live birth rates are required to validate these findings and develop targeted therapeutic strategies.

## 5. Conclusions

This study establishes dysregulated protein acetylation as a defining molecular signature of idiopathic male infertility (IMI). Global acetyl-proteomic profiling revealed key hub protein alterations in antioxidant defense (SOD1, PARK7), energy metabolism (PRKACA), and spermatogenesis pathways. Aberrant acetylation of these proteins is likely to disturb redox homeostasis, impair mitochondrial function, and compromise sperm motility and fertilizing potential, thereby contributing to the pathophysiology of IMI. These findings underscore lysine acetylation as a critical regulatory mechanism in sperm physiology and a promising source of diagnostic biomarkers and therapeutic targets. The development of clinically applicable, acetylation-based biomarker panels could facilitate precision diagnostics and personalized treatment strategies for male infertility. Future multicenter, longitudinal studies correlating these molecular signatures with fertilization, embryo development, and live birth outcomes will be essential to validate their clinical relevance and translational potential.

## Figures and Tables

**Table 1:** Ingenuity Pathway Analysis (IPA) of differentially acetylated proteins between idiopathic infertile patients (IIP) and fertile donors (FD). The table lists top enriched functional categories, associated *p*-values (≤0.00001), activation z-scores, and molecules involved, highlighting dysregulation of redox homeostasis, antioxidant defense, and reproductive system disease pathways, including asthenozoospermia.

Categories	Diseases or Functions Annotation	*p*-Value	Activation z-Score	Proteins
Free Radical Scavenging, Molecular Transport	Quantity of reactive oxygen species	0.00000081	1.66	AIFM1, AKR1B1, ALOX15B, APOE, APP, AQP7, ATP5PO, BCL2L1, BPNT2, CAT, DCD, EEF1G, EPHX1, FTH1, GLO1, GPX4, GSR, GSTP1, HSPA4, HSPA9, KRAS, LCN2, PARK7, PFN1, PGD, PRDX6, PREP, PRKDC, PSEN2, SOD1, UQCRFS1, VDAC3
Organismal Injury and Abnormalities, Reproductive System Disease	Infertility and Asthenozoospermia	0.0000231	1.387	AKAP4, ARHGDIA, ATP1A4, ATP2B4, BGN, BSG, CA4, CFAP52, DPY19L2, ELAPOR1, H1-6, HSD17B4, HSPA4, IGF2R, KRAS, KRT8, LDHC, MNS1, NECTIN2, PITHD1, PIWIL1, PRKACA, PRKRA, RAD23B, SEPTIN4, SOD1, SPAG6, TALDO1, TEX101, TEX14, VDAC3, YWHAE, ZPBP

## Data Availability

The data that support the findings of this study are available from the corresponding authors upon reasonable request.

## Supplementary Materials

The following supporting information can be downloaded at: https://www.mdpi.com/article/10.3390/antiox14121410/s1, Table S1: Semen parameters in Control (fertile donors) and idiopathic infertile patients (IIP), Table S2: List of 61 spermatozoa lysine acetylated proteins associated with idiopathic infertile patients with respect to control (fertile donors).

## Abbreviations

The following abbreviations are used in this manuscript:

NOA	Non-obstructive azoospermia
IMI	Idiopathic male infertility
ROS	Reactive oxygen species
DEPs	Differentially expressed proteins
IPA	Ingenuity pathway analysis
GO	Gene ontology
KEGG	Kyoto Encyclopedia of Genes and Genomes

## References

[1] Goutami L., Jena S.R., Moharana A.K., Pradhan A., Kar S., Samanta L. (2025). HSPA2 emerges as a key biomarker: Insights from global lysine acetylproteomic profiling in idiopathic male infertility. Cell Stress Chaperones.

[2] Gosalvez J., Tvrda E., Agarwal A. (2017). Free radical and superoxide reactivity detection in semen quality assessment: Past, present, and future. J. Assist. Reprod. Genet..

[3] Wang Z., Li D., Zhou G., Xu Z., Wang X., Tan S., Li Z., Li X., Song C., Yuan S. (2025). Deciphering the role of reactive oxygen species in idiopathic asthenozoospermia. Front. Endocrinol..

[4] Dias T.R., Samanta L., Agarwal A., Pushparaj P.N., Panner Selvam M.K., Sharma R. (2019). Proteomic signatures reveal differences in stress response, antioxidant defense and proteasomal activity in fertile men with high seminal ROS levels. Int. J. Mol. Sci..

[5] O’Flaherty C. (2014). Peroxiredoxins: Hidden players in the antioxidant defence of human spermatozoa. Basic Clin. Androl..

[6] Shah T., Shin D. (2020). Empiric medical therapy for idiopathic male infertility. Male Infertility: Contemporary Clinical Approaches, Andrology, ART and Antioxidants.

[7] Shrivastava N., Shrivastava V., Pandey M. (2021). A Study of Role of Reactive Oxygen Species in Idiopathic Male Infertility & Its Management by Antioxidant Therapy in Uttar Pradesh (UP). Evid. Based Med. Healthc..

[8] Kaur M., Ghosal A., Kaur R., Chhabra K., Kapoor H.S., Khetarpal P. (2024). Exposure to potentially toxic elements (PTEs) and the risk of male infertility-A Systematic review and meta-analysis. J. Gynecol. Obstet. Hum. Reprod..

[9] Ottolenghi S., Rubino F.M., Sabbatini G., Coppola S., Veronese A., Chiumello D., Paroni R. (2019). Oxidative stress markers to investigate the effects of hyperoxia in anesthesia. Int. J. Mol. Sci..

[10] Liu P., Xiao J., Wang Y., Song X., Huang L., Ren Z., Kitazato K., Wang Y. (2021). Posttranslational modification and beyond: Interplay between histone deacetylase 6 and heat-shock protein 90. Mol. Med..

[11] Guo Z., Ma X., Zhang R.X., Yan H. (2023). Oxidative stress, epigenetic regulation and pathological processes of lens epithelial cells underlying diabetic cataract. Adv. Ophthalmol. Pract. Res..

[12] Sharma R., Agarwal A., Mohanty G., Hamada A.J., Gopalan B., Willard B., Yadav S., Du Plessis S. (2013). Proteomic analysis of human spermatozoa proteins with oxidative stress. Reprod. Biol. Endocrinol..

[13] Clough T., Thaminy S., Ragg S., Aebersold R., Vitek O. (2012). Statistical protein quantification and significance analysis in label-free LC-MS experiments with complex designs. BMC Bioinform..

[14] WHO (2010). WHO Laboratory Manual for the Examination and Processing of Human Semen.

[15] Sharma R., Biedenharn K.R., Fedor J.M., Agarwal A. (2013). Lifestyle factors and reproductive health: Taking control of your fertility. Reprod. Biol. Endocrinol..

[16] Zhou Y., Zhang H., Yan H., Han P., Zhang J., Liu Y. (2025). Deciphering the Role of Oxidative Stress in Male Infertility: Insights from Reactive Oxygen Species to Antioxidant Therapeutics. Front. Biosci. Landmark.

[17] Panner Selvam M.K., Samanta L., Agarwal A. (2020). Functional analysis of differentially expressed acetylated spermatozoal proteins in infertile men with unilateral and bilateral varicocele. Int. J. Mol. Sci..

[18] Zhao Q., Zhang Z., Li J., Xu F., Zhang B., Liu M., Liu Y., Chen H., Yang J., Zhang J. (2020). Lysine acetylome study of human hepatocellular carcinoma tissues for biomarkers and therapeutic targets discovery. Front. Genet..

[19] Yu H., Diao H., Wang C., Lin Y., Yu F., Lu H., Xu W., Li Z., Shi H., Zhao S. (2015). Acetylproteomic analysis reveals functional implications of lysine acetylation in human spermatozoa (sperm). Mol. Cell. Proteom..

[20] Marques C., Guo W., Pereira P., Taylor A., Patterson C., Evans P.C., Shang F. (2006). The triage of damaged proteins: Degradation by the ubiquitin-proteasome pathway or repair by molecular chaperones. FASEB J. Off. Publ. Fed. Am. Soc. Exp. Biol..

[21] Zhang Y., Wang S., Zhang L., Zhou F., Zhu K., Zhu Q., Liu Q., Liu Y., Jiang L., Ning G. (2020). Protein acetylation derepresses serotonin synthesis to potentiate pancreatic beta-cell function through HDAC1-PKA-Tph1 signaling. Theranostics.

[22] Nixon B., Johnston S.D., Skerrett-Byrne D.A., Anderson A.L., Stanger S.J., Bromfield E.G., Martin J.H., Hansbro P.M., Dun M.D. (2019). Modification of crocodile spermatozoa refutes the tenet that post-testicular sperm maturation is restricted to mammals. Mol. Cell. Proteom..

[23] Pryor W.A., Houk K.N., Foote C.S., Fukuto J.M., Ignarro L.J., Squadrito G.L., Davies K.J. (2006). Free radical biology and medicine: It’s a gas, man!. Am. J. Physiol. Regul. Integr. Comp. Physiol..

[24] Carracedo S., Briand-Amirat L., Dordas-Perpinyà M., Escuredo Y.R., Delcombel R., Sergeant N., Delehedde M. (2022). ProAKAP4 protein marker: Towards a functional approach to male fertility. Anim. Reprod. Sci..

[25] du Plessis S.S., Agarwal A., Mohanty G., Van der Linde M. (2015). Oxidative phosphorylation versus glycolysis: What fuel do spermatozoa use?. Asian J. Androl..

[26] Agarwal A., Leisegang K., Majzoub A., Henkel R., Finelli R., Selvam M.K.P., Tadros N., Parekh N., Ko E.Y., Cho C.-L. (2021). Utility of antioxidants in the treatment of male infertility: Clinical guidelines based on a systematic review and analysis of evidence. World J. Men’s Health.

[27] Aitken R.J., Drevet J.R. (2020). The importance of oxidative stress in determining the functionality of mammalian spermatozoa: A two-edged sword. Antioxidants.

[28] Bach H.A., Vu P.N., Ma T.H.T., Nguyen H.H., Tran Duc P., Bui Minh D., Nong V.H., Nguyen D.T. (2023). Genetic variations of antioxidant genes and their association with male infertility in Vietnamese men. J. Clin. Lab. Anal..

[29] Yan L., Liu J., Wu S., Zhang S., Ji G., Gu A. (2014). Seminal superoxide dismutase activity and its relationship with semen quality and SOD gene polymorphism. J. Assist. Reprod. Genet..

[30] Turnham R.E., Scott J.D. (2016). Proteinkinase A catalytic subunit isoform PRKACA.; History, function and physiology. Gene.

[31] Zapata-Carmona H., Barón L., Kong M., Morales P. (2021). Protein kinase a (PRKA) activity is regulated by the proteasome at the onset of human sperm capacitation. Cells.

[32] Skou L.D., Johansen S.K., Okarmus J., Meyer M. (2024). Pathogenesis of DJ-1/PARK7-mediated Parkinson’s disease. Cells.

[33] Hosseini M., Khalafiyan A., Zare M., Karimzadeh H., Bahrami B., Hammami B., Kazemi M. (2024). Sperm epigenetics and male infertility: Unraveling the molecular puzzle. Hum. Genom..

[34] Wang Y., Fu X., Li H. (2025). Mechanisms of oxidative stress-induced sperm dysfunction. Front. Endocrinol..

[35] Bouhadana D., Godin Pagé M.-H., Montjean D., Bélanger M.-C., Benkhalifa M., Miron P., Petrella F. (2025). The Role of Antioxidants in Male Fertility: A Comprehensive Review of Mechanisms and Clinical Applications. Antioxidants.

[36] Mojica-Villegas M.A., Izquierdo-Vega J.A., Chamorro-Cevallos G., Sánchez-Gutiérrez M. (2014). Protective effect of resveratrol on biomarkers of oxidative stress induced by iron/ascorbate in mouse spermatozoa. Nutrients.

[37] Santonastaso M., Mottola F., Iovine C., Colacurci N., Rocco L. (2021). Protective effects of curcumin on the outcome of cryopreservation in human sperm. Reprod. Sci..

[38] Çolak D.A., Uysal H. (2017). Protective effects of coenzyme Q10 and resveratrol on oxidative stress induced by various dioxins on transheterozigot larvae of Drosophila melanogaster. Toxicol. Res..

[39] Agarwal A., Panner Selvam M.K., Samanta L., Vij S.C., Parekh N., Sabanegh E., Tadros N.N., Arafa M., Sharma R. (2019). Effect of antioxidant supplementation on the sperm proteome of idiopathic infertile men. Antioxidants.

