# Supplementary material for: Spermatozoa Proteins Involved in ROS Generation and Antioxidant Defense Are Differentially Acetylated in Idiopathic Infertility
Source: Antioxidants (Basel). 2025 Nov 26;14(12):1410. doi: 10.3390/antiox14121410 (PMC12729651; doi:10.3390/antiox14121410)
Supplement: Supplementary file 1 [file antioxidants-14-01410-s001.zip › antioxidants-3959834-supplementary.pdf]

Supplementary Tables

Table S1. Semen parameters in Control (fertile donors) and idiopathic infertile patients (IIP).

Parameter	WHO	Fertile (N=43)	Infertile (N=57)	p Value
Volume (mL)	> 1.5	3.23 ± 0.61	3.1 ± 0.69	>0.05
pH	7.2–8.0	7.31 ± 0.18	7.34 ± 0.11	>0.05
Sperm Motility (%)	> 40	51.98 ± 10.89	37.89 ± 9.57	p < 0.05
Sperm Concentration (Million/ml)	> 15	122.46 ± 26.57	88.65 ± 39.29	p < 0.05
Sperm Viability (%)	> 58	66.78 ± 8.19	53.57 ± 10.21	p < 0.05
Sperm Morphology (%)	> 4	85.2 ± 7.94	78.12 ± 20.71	p < 0.05
Age	< 35	28.86 ± 4.19	30.54 ± 4.54	p = 0.1201

Table S2. List of 61 spermatozoa lysine acetylated proteins associated with idiopathic infertile patients with respect to control (fertile donors).

Sl. No.	Protein FDR Confidence: Combined	Accession	Gene Name	log2 fold Change	pValue
**1.**	High	P52209	PGD	4.256191762	5.94E-05
**2.**	High	Q96J94	PIWIL1	2.679191115	0.00060977
**3.**	High	P22492	H1-6	2.492856646	0.00095937
**4.**	High	P48147	PREP	2.440087522	0.00156825
**5.**	High	O14520	AQP7	2.341455425	0.000455379
**6.**	High	Q6NUT2	DPY19L2	1.770550027	0.007592917
**7.**	High	Q13733	ATP1A4	1.667362297	0.008208244
**8.**	Medium	Q8IWB6	TEX14	1.641381853	0.043911425
**9.**	High	Q9Y277	VDAC3	1.485430776	0.012078702
**10.**	High	P23634	ATP2B4	1.359640946	0.01646376
**11.**	High	Q6UXG2	ELAPOR1	1.253302509	0.030530666
**12.**	High	P80188	LCN2	1.201902045	0.017077919
**13.**	High	Q5JQC9	AKAP4	1.078397486	0.037360737
**14.**	High	P48047	ATP5PO	0.946286444	0.023437074

**15.**	High	P78527	PRKDC	0.886882395	0.015038486
**16.**	High	P04040	CAT	0.874561034	0.048875111
**17.**	High	P36969	GPX4	0.719660988	0.014290688
**18.**	High	P51659	HSD17B4	0.663237791	0.022398858
**19.**	High	P21810	BGN	0.642886633	0.023653858
**20.**	High	P02794	FTH1	0.551903055	0.027889294
**21.**	High	P35613	BSG	0.520217415	0.025507442
**22.**	High	P15121	AKR1B1	0.345672629	0.04762866
**23.**	High	P38646	HSPA9	0.19972737	0.047144885
**24.**	High	Q9NX62	BPNT2	0.157444518	0.04800982
**25.**	High	Q92692	NECTIN2	0.036165141	0.049469507
**26.**	High	P47985	UQCRFS1	-0.01140692	0.049852308
**27.**	Medium	Q07817	BCL2L1	-0.083762393	0.049154978
**28.**	High	P49810	PSEN2	-0.118559887	0.048365341
**29.**	High	O75569	PRKRA	-0.129501919	0.048164826
**30.**	High	P17612	PRKACA	-0.204778381	0.046927824
**31.**	High	O75602	SPAG6	-0.272529706	0.045665432
**32.**	High	P34932	HSPA4	-0.287828032	0.015262641
**33.**	High	P22748	CA4	-0.294390936	0.015700549
**34.**	High	Q9BS86	ZPBP	-0.341021333	0.045273658
**35.**	High	P07737	PFN1	-0.393384325	0.041585799
**36.**	High	O15296	ALOX15B	-0.39468525	0.04827126
**37.**	High	Q9BY14	TEX101	-0.421807214	0.039900991
**38.**	High	P09211	GSTP1	-0.475504682	0.036877605
**39.**	High	Q04760	GLO1	-0.622049078	0.024320173
**40.**	High	P11717	IGF2R	-0.684854644	0.014229879
**41.**	High	Q9GZP4	PITHD1	-0.712644453	0.025322497
**42.**	High	P30041	PRDX6	-0.717601444	0.016777685

**43.**	High	P07099	EPHX1	-0.74049007	0.024232458
**44.**	High	Q8N1V2	CFAP52	-0.966317579	0.010146135
**45.**	High	P62258	YWHAE	-1.052902618	0.0406287
**46.**	High	P00390	GSR	-1.089511235	0.046711246
**47.**	High	P01116	KRAS	-1.139539421	0.040159516
**48.**	High	P05067	APP	-1.148682523	0.013785452
**49.**	High	P52565	ARHGDIA	-1.169828737	0.041209324
**50.**	High	P02649	APOE	-1.187998035	0.046650134
**51.**	High	P81605	DCD	-1.307503814	0.020469942
**52.**	High	P54727	RAD23B	-1.428189538	0.038895801
**53.**	High	P26641	EEF1G	-1.617545386	0.01574704
**54.**	High	P37837	TALDO1	-1.644965601	0.019575986
**55.**	High	O95831	AIFM1	-1.650273384	0.020314328
**56.**	High	P05787	KRT8	-2.305379748	0.01166444
**57.**	High	Q8NEH6	MNS1	-2.355083297	0.040525886
**58.**	High	O43236	SEPTIN4	-2.516527485	0.00324634
**59.**	High	P07864	LDHC	-2.752456425	0.003187312
**60.**	High	P00441	SOD1	-2.917318536	0.019394523
**61.**	High	Q99497	PARK7	-4.323231262	3.11E-05
